# Sjögren's Syndrome: Animal Models, Etiology, Pathogenesis, Clinical Subtypes, and Diagnosis

**DOI:** 10.1155/2019/8101503

**Published:** 2019-05-20

**Authors:** Long Shen, Jing He, Jill M. Kramer, Vatinee Y. Bunya

**Affiliations:** ^1^University at Buffalo, Buffalo, USA; ^2^Peking University People's Hospital, Beijing, China; ^3^Scheie Eye Institute, University of Pennsylvania, Philadelphia, USA

Sjögren's syndrome (SS) is a chronic autoimmune disease that classically affects the lacrimal and salivary glands resulting in dry eyes and dry mouth and can affect almost any organ system in the body including the lungs, kidney, and central nervous system [1]. Many systemic aspects of SS are described, including B cell lymphoma in addition to pulmonary and renal pathoses [2–4]. Diagnosis often takes several years once symptoms manifest [5], and even after the diagnosis is rendered, there are currently no treatments available that address the underlying disease etiology.

While the pathogenesis of SS is not well understood, both innate and adaptive immune responses are implicated in disease initiation and progression. In the innate response, an antiviral response is mounted through the recognition of viral nucleic acids by Toll-like receptors (TLRs). This recognition leads to the upregulation of the type 1 interferon (IFN) pathway. However, the means by which immune activation is initiated and maintained remain incompletely understood. Activation of TLRs is critical for the progression of numerous autoimmune diseases [6], although there are relatively few studies regarding the role of these receptors in SS. Studies in mice and humans reveal that TLRs are potent mediators of inflammation in SS. TLRs are expressed and functional in salivary tissue, and TLRs in peripheral blood cells of SS patients are also upregulated and hyperresponsive to ligation. In addition, interferon signatures have been detected in the blood and in the labial salivary gland tissues of patients with pSS [7]. However, it is unclear whether TLRs are activated by microbial products or host-derived ligands in SS [8]. Both animal models and patient studies are instrumental in understanding causes of TLR dysfunction in SS [9]. Studies that identify specific TLRs and clinically relevant ligands will likely lead to the development of novel therapeutics that will diminish chronic inflammation that is a characteristic of SS.

Several viruses have been implicated as possible environmental triggers of SS including Epstein-Barr virus (EBV), cytomegalovirus (CMV), human herpes virus type 8 (HHV-8), human T-lymphotropic virus type 1 (HTLV-1), hepatitis C virus, and enterovirus [10]. In addition, paramyxovirus, which causes mumps, may persist in salivary glands and could provide the necessary trigger to initiate autoimmunity pathogenesis of SS in certain genetically susceptible individuals [11]. Cytokines, B cell growth factor, and serum B cell-activating factor (BAFF) levels also play an important role in the pathogenesis of SS. They are altered in patients with SS and correlate with antibodies to Ro and La [12, 13]. Previous studies have shown that most IL-14*α* transgenic mice develop gastrointestinal B cell lymphomas [14].

Most often, SS presents with polymorphic clinical presentations including the involvement of various systemic organs, which can result in delays in diagnosis. While SS is typically not associated with significant mortality, patients who demonstrate renal involvement are at risk for life-threatening complications. Renal involvement in SS is relatively rare and is seen in approximately 5-10% of SS patients [15–18]. Studies are needed to understand the mechanisms that govern kidney dysfunction in SS and to uncover the optimal diagnostic strategies for SS patients with renal complications. In addition, it is important to identify SS patients who are at high risk of renal disease in SS in order to manage these individuals appropriately.

Non-Hodgkin lymphoma is another serious systemic manifestation of SS [19, 20]. SS is characterized by B cell hyperactivity, and the lymphomas that arise in SS patients originate exclusively from B cells [21]. There are several clinical and serological findings that are associated with lymphoma development in SS patients, including anemia, neutropenia, and thrombocytopenia [22]. In addition, BAFF polymorphisms have been identified, and BAFF levels tend to be elevated in patients who develop lymphoma [12, 23–25]. While the relationship between lymphoma development, BAFF levels, and autoantibodies is not well understood, further work is needed to identify autoantibodies that may be indicative or predictive of lymphoma development in SS [21].

The diagnosis of SS can be very challenging due to the absence of specific clinical manifestations in the early stages of the disease and the lack of noninvasive diagnostic methods with high specificity and sensitivity. This can lead to significant delays in treatment and worse clinical outcomes. Therefore, novel biomarkers and imaging methods to help recognize the disease at an early stage are needed. For example, antibodies to salivary gland protein 1 (SP1), carbonic anhydrase 6 (CA6), and parotid secretory protein autoantibodies (PSP) were first described in a mouse model for SS and have also been found in SS patients together and without anti-SSA/Ro, as well as in patients with idiopathic dry mouth and dry eye disease [26]. However, despite the discovery of new biomarkers such as these, there are continued diagnostic delays due to the limited understanding of the sequence of events that trigger the activation of the autoimmunity against specific antigenic targets. It is important to study and elucidate the pathways leading to the activation of the antigenic targets as it may lead to more precise disease diagnosis and to the development of specific targeted therapies.

The ocular manifestations of SS often cause substantial morbidity as patients experience significant decreases in quality of life and visual functioning as a result of the disease [27]. Approximately 10% of dry eye patients have underlying SS, but because dry eye is a highly prevalent condition in the general population [27], it is not possible to work-up all dry eye patients for SS. Therefore, better tests that distinguish SS from other causes of dry eye are needed. In addition, further work is required to understand the inciting events that lead to the development of the ocular manifestations of SS. While immune dysfunction is thought to underlie the ocular dysfunction observed, [28] the contribution of environmental insults and genetics to the disease remain incompletely understood [29]. Thus, further work to understand the mechanisms that govern ocular dysfunction and to develop tests for dry eye that identify patients with SS will have a significant impact.

The goal of this special issue is to highlight recent advances in the understanding of the etiopathogenesis, varying clinical presentations, and diagnosis of SS. For example, Z. Mackiewicz et al. in their article entitled “Sjögren's Syndrome: Concerted Triggering of Sicca Conditions” evaluate the role of the paramyxovirus in SS. J. Kramer and J. Kiripolsky in their paper “Current and Emerging Evidence for Toll-Like Receptor Activation in Sjögren's Syndrome” explore the role of TLRs in the pathogenesis of SS based on findings in the various mouse models. They discuss the role and significance of putative damage-associated molecular patterns in SS. J. Luo et al. discuss renal involvement of SS, its diverse clinical presentation, and the role of renal biomarkers in kidney damage assessment in their article “High-Risk Indicators of Renal Involvement in Primary Sjogren's Syndrome: A Clinical Study of 1002 Cases.” Z. Xian and colleagues, in their study entitled “Association between B Cell Growth Factors and Primary Sjögren's Syndrome-Related Autoantibodies in Patients with Non-Hodgkin's Lymphoma,” describe the relation between the cytokines BAFF and IL-14 with various traditional autoantibodies (anti-SSA/Ro) and newer tissue-specific autoantibodies. They also describe the role of cytokines and autoantibodies for the stratification of SS patients with gastrointestinal lymphomas. Finally, S. Karakus and colleagues conducted a cross-sectional study of dry eye patients to investigate the clinical significance of anti-salivary gland protein 1 (SP1), anti-carbonic anhydrase 6 (CA6), and anti-parotid secretory protein (PSP) autoantibodies. In their manuscript “Clinical Correlations of Novel Autoantibodies in Patients with Dry Eye,” they demonstrate that disease severity stratification may be feasible using new biomarkers and conclude that anti-CA6 is seen in patients with severe aqueous-deficient dry eye. Taken together, it appears that a new era is on the horizon for a better understanding of the clinical manifestations, diagnosis, etiology, and pathogenesis of SS.
